# Improving estimates of pertussis burden in Ontario, Canada 2010–2017 by combining validation and capture-recapture methodologies

**DOI:** 10.1371/journal.pone.0273205

**Published:** 2023-12-01

**Authors:** Shilo H. McBurney, Jeffrey C. Kwong, Kevin A. Brown, Frank Rudzicz, Andrew Wilton, Natasha S. Crowcroft

**Affiliations:** 1 Dalla Lana School of Public Health, University of Toronto, Toronto, Ontario, Canada; 2 Department of Epidemiology, Brown University School of Public Health, Providence, Rhode Island, United States of America; 3 Public Health Ontario, Toronto, Ontario, Canada; 4 Department of Laboratory Medicine and Pathobiology, University of Toronto, Toronto, Ontario, Canada; 5 ICES, Toronto, Ontario, Canada; 6 Department of Family & Community Medicine, University of Toronto, Toronto, Ontario, Canada; 7 Department of Computer Science, University of Toronto, Toronto, Ontario, Canada; 8 Faculty of Computer Science, Dalhousie University, Halifax, Nova Scotia, Canada; 9 Vector Institute for Artificial Intelligence, Toronto, Ontario, Canada; 10 Immunization, Vaccines and Biologicals, World Health Organization, Geneva, Switzerland; Universidade Federal de Minas Gerais, BRAZIL

## Abstract

An underestimation of pertussis burden has impeded understanding of transmission and disallows effective policy and prevention to be prioritized and enacted. Capture-recapture analyses can improve burden estimates; however, uncertainty remains around incorporating health administrative data due to accuracy limitations. The aim of this study is to explore the impact of pertussis case definitions and data accuracy on capture-recapture estimates. We used a dataset from March 7, 2010 to December 31, 2017 comprised of pertussis case report, laboratory, and health administrative data. We compared Chao capture-recapture abundance estimates using prevalence, incidence, and adjusted false positive case definitions. The latter was developed by removing the proportion of false positive physician billing code-only case episodes after validation. We calculated sensitivity by dividing the number of observed cases by abundance. Abundance estimates demonstrated that a high proportion of cases were missed by all sources. Under the primary analysis, the highest sensitivity of 78.5% (95% CI 76.2–80.9%) for those less than one year of age was obtained using all sources after adjusting for false positives, which dropped to 43.1% (95% CI 42.4–43.8%) for those one year of age or older. Most code-only episodes were false positives (91.0%), leading to considerably lower abundance estimates and improvements in laboratory testing and case report sensitivity using this definition. Accuracy limitations can be accounted for in capture-recapture analyses using different case definitions and adjustment. The latter enhanced the validity of estimates, furthering the utility of capture-recapture methods to epidemiological research. Findings demonstrated that all sources consistently fail to detect pertussis cases. This is differential by age, suggesting ascertainment and testing bias. Results demonstrate the value of incorporating real time health administrative data into public health surveillance if accuracy limitations can be addressed.

## Introduction

Pertussis remains one of the most common vaccine-preventable diseases in Canada [1]. In 2019, the last year before the Covid-19 pandemic, the reported incidence of pertussis in Canada was 5.64 per 100,000 [1]. Despite being a reportable disease, an underestimation of cases and deaths has impeded understanding of transmission. Canada relies on a passive surveillance system for pertussis, with local public health units responsible for reporting cases to provincial health authorities that were in turn reported from laboratories, health practitioners, hospital administrators, schools, and other institutions [2]. As a result, ascertainment bias is a key concern. This occurs when atypical cases are underdiagnosed, including older individuals experiencing mild disease [3, 4]. This issue is worsened by testing bias, with younger, severe cases more likely to be tested, have a positive result, and be reported [5, 6]. It is suspected that milder, undetected cases may be sustaining pertussis transmission in Canadian communities [4].

Burden estimates vary regionally based on interactions between case definitions, the type of surveillance and data available, practitioner knowledge, immunization programs, and the extent of local transmission [5, 7]. Underestimation may be attributed to failing to consider pertussis diagnostically, atypical presentations, infrequent diagnostic testing, suboptimal test accuracy, lack of uniformity in case definitions, and reporting issues [3, 6]. In combination with complicated epidemiological characteristics, pertussis surveillance is consequently challenging [5, 8]. However, monitoring burden is essential for informing and assessing the impact of immunization programs and policy [5, 7–10].

To improve pertussis burden estimates, one strategy is to supplement surveillance data with health administrative data [11]. When several sources are available, capture-recapture analyses can be used to better estimate burden [12]. This analytic approach has been recently used to assess completeness of contact-tracing for Ebola and detection of Covid-19 infections [13, 14]. For pertussis, capture-recapture has been used to estimate the number of deaths in England and the number of cases in Ontario, Canada [11, 15]. The latter study estimated that 21–73% of cases have been missed using combined surveillance, laboratory, and health administrative data [11]. However, considerable uncertainty in estimation remained around the validity of using health administrative data, and particularly Ontario Health Insurance Plan (OHIP) physician billing diagnostic codes. The aim of this study is to evaluate the impact of using different pertussis case definitions on capture-recapture estimates including abundance and data source sensitivity, with the goal of enhancing the utility of this method for improving burden estimates to inform public health surveillance, prevention, and policy.

## Methods

The University of Toronto’s Health Sciences Research Ethics Board (37885) and the Public Health Ontario (PHO) Ethics Review Board (2019–006.02) approved this study. The need for consent was waived by the ethics committees. Data were linked and analyzed at ICES (formerly the Institute of Clinical Evaluative Sciences) using unique encoded identifiers. The dataset from this study is held securely in coded form at ICES. While legal data sharing agreements between ICES and data providers (e.g., healthcare organizations and government) prohibit ICES from making the dataset publicly available, access may be granted to those who meet pre-specified criteria for confidential access, available at https://www.ices.on.ca/DAS (email: das@ices.on.ca).

### Data sources

We obtained Public Health Information System (iPHIS) and PHO Laboratory Information System (Labware) data from a PHO linked dataset previously used to improve estimates of pertussis burden in Ontario [11]. iPHIS data contained confirmed, probable, and “does not meet” (DNM) pertussis case reports. We considered reports greater than 365 days apart a new case, with data available from April 1, 2006 to March 31, 2015. When duplicates occurred in the same year, we gave priority to the highest level of confirmation. Labware data included positive, indeterminate, and negative pertussis laboratory tests, with cases defined as at least one positive result by PCR or culture. We counted positive results more than 90 days apart as a new case, and data were available from December 7, 2009 to March 31, 2015. Time intervals for data extraction were selected due to the availability of each data source, with the study period limited by Labware data collection beginning in 2009. iPHIS entries prior to December 1, 2009 were excluded accordingly (n = 3464).

We updated the PHO dataset until March 31, 2018 and combined it with health administrative data from December 1, 2009 from three databases held at ICES: the Canadian Institute for Health Information (CIHI) Discharge Abstract Database (DAD); the CIHI National Ambulatory Care Reporting System (NACRS); and the Ontario Health Insurance Plan (OHIP) Claims Database (Table 1). Labware and iPHIS entries that were unable to link to a unique ICES key number (IKN) after using both deterministic and probabilistic linkage methods were excluded (n = 1020). Collected health administrative data included ICD-10 codes A37.0 (whooping cough, *Bordetella pertussis)* and A37.9 (whooping cough, unspecified species) from hospitalizations and emergency room visits and OHIP diagnostic billing code 033 (whooping cough, *Bordetella pertussis*). We restricted OHIP claims to billings from homes, offices, and long-term care facilities. The Registered Persons Database (RPDB) was used to obtain data on patient sex, age, and date of death. We excluded health administrative data entries with same-day immunizations (n = 8305) as they are unlikely to reflect a true pertussis case. To do so, we used identified pertussis data entries with pertussis-containing immunization codes G840, G841, and G847 and general immunization codes G538 and G539 documented on the same day. We also excluded same-day duplicate entries for individuals (n = 260), as only one entry a day at maximum would reflect a true pertussis case. We excluded all entries with no index date or a date of death prior to the index date, as well as participants with an invalid unique identifier or who were missing their sex or birth date (n = 471).

**Table 1:** Initial sample and final sample post-exclusion criteria by pertussis data source.

Data Source		Collection Start Date	Collection End Date	Number of Entries (%)	
**Initial sample (N = 112,541)**					
iPHIS		April 1, 2006	March 31, 2018	7827 (7.0)	
Labware		December 7, 2009		33,740 (30.0)	
Health administrative data		December 1, 2009		70,947 (63.0)	
**Final sample (n = 98,994)**					
iPHIS		December 1, 2009	March 31, 2018	4026 (4.07)	
	*Confirmed cases*				2879 (2.91)
	*Probable cases*				537 (0.54)
	*Does not meet case definitions*				610 (0.62)
Labware		December 7, 2009		32,608 (32.9)	
	*Positive laboratory results*				2804 (2.83)
	*Indeterminate laboratory results*				810 (0.82)
	*Negative laboratory results*				29,714 (30.0)
Health administrative data		December 1, 20		63,360 (63.0)	
	*DAD hospitalizations alone*				352 (0.36)
	*NACRS emergency visits alone*				5872 (5.93)
	*OHIP physician billings alone*				55,889 (56.5)
	*Any combination of DAD or NACRS or OHIP*				247 (0.25)

### Case definitions and sensitivity analyses

We developed three case definitions to separately input into capture-recapture models–period prevalence, incidence with exclusions, and false-positive adjusted (Table 2). Period prevalence permitted an individual to have one entry per data source over the study period. We did not apply a time limit to recapture in other sources, leading to the best recapture scenario. For incidence with exclusions, we incorporated data entries into episodes using 90-day rules and ruled out administrative data-only episodes with a negative pertussis laboratory test within 28 days of the episode start (Table 2 and S1 Fig). This structure required recapture in other sources to occur within the 90 days before or after the respective episode. Finally, for the last definition we eliminated the proportion of false positive OHIP code-only episodes from the incidence with exclusions case definition. To do so, we validated these episodes to obtain a positive predictive value (PPV) and took a random sample based on the estimated proportion of true positives. We conducted validation using a previously developed methodology and cohort from the Electronic Medical Record Primary Care (EMRPC) database (S1 Appendix) [16, 17]. The PPV was estimated as 8.99%, leading to the removal of 41,062 OHIP-code only entries for the primary analysis.

**Table 2:** Descriptions of case definitions and number of observed pertussis cases by analysis.

Case Definition	Description	Analysis^Ϯ^		Observed cases
**Period prevalence**	Individuals were only permitted to have one entry per data source over the study period. Recapture could occur at any time over the study period.	Primary analysis		48,931
			*< 1 year of age*	2636
			*≥ 1 year of age*	46,295
		Sensitivity analysis 1		49,229
			*< 1 year of age*	2648
			*≥ 1 year of age*	46,581
		Sensitivity analysis 2		49,986
			*< 1 year of age*	2729
			*≥ 1 year of age*	47,257
		Sensitivity analysis 3		44,496
			*< 1 year of age*	2050
			*≥ 1 year of age*	42,446
**Incidence with exclusions**	Entries were incorporated into episodes using 90-day rules, after which administrative data-only episodes were ruled out in cases with a negative pertussis laboratory test within 28 days of the episode start. Recapture had to occur within 90 days before or after the respective episode.	Primary analysis		50,794
			*< 1 year of age*	2118
			*≥ 1 year of age*	48,676
		Sensitivity analysis 1		51,148
			*< 1 year of age*	2133
			*≥ 1 year of age*	49,015
		Sensitivity analysis 2		51,757
			*< 1 year of age*	2206
			*≥ 1 year of age*	49,551
		Sensitivity analysis 3		48,274
			*< 1 year of age*	1863
			*≥ 1 year of age*	46,411
**Adjusted false positives**	Incidence with exclusion episodes where 91.01% of OHIP code-only episodes were removed after identifying the proportion of false positive OHIP code-only episodes through validation.	Primary analysis		9732
			*< 1 year of age*	904
			*≥ 1 year of age*	8828
		Sensitivity analysis 1		10,192
			*< 1 year of age*	897
			*≥ 1 year of age*	9295
		Sensitivity analysis 2		10,924
			*< 1 year of age*	1007
			*≥ 1 year of age*	9917
		Sensitivity analysis 3		7167
			*< 1 year of age*	626
			*≥ 1 year of age*	6541

^Ϯ^Primary analysis = includes confirmed iPHIS case reports, Labware positive laboratory tests, and health administrative data, Sensitivity analysis 1 = additionally included probable iPHIS case reports, Sensitivity analysis 2 = additionally included iPHIS probable and “does not meet” case reports and Labware indeterminate laboratory tests, Sensitivity analysis 3 = additionally excluded A37.9 codes (pertussis species unspecified)

To ensure that failure to recapture was not due to data missingness, we applied a study window based on data availability. We removed cases with a first date before March 7, 2010 and last date after December 31, 2017 to give a buffer of 90 days at each end to ensure an accurate episode start and end date, which we defined as the earliest and latest date for an episode in any data source. For the primary analysis, we included iPHIS confirmed cases, Labware positive PCR or culture results, and administrative data. However, we applied three sensitivity analyses to the three data structures described above to explore further uncertainty in case definitions. For the first sensitivity analysis, we incorporated iPHIS probable cases (n = 537). iPHIS probable and DNM cases were included in the second sensitivity analysis in addition to indeterminate laboratory tests (n = 1957). Episodes were also ruled out if the episode only included administrative data or DNM entries and there was a negative pertussis laboratory test within 28 days of the episode start, reflecting a possible false positive entry. For the final sensitivity analysis, we excluded entries with A37.9 ICD-10 codes (n = 5749), meaning the pertussis species was unspecified. For each sensitivity analysis, the new datasets with entries included or excluded were input into the different case definition structures prior to capture-recapture analysis.

### Capture-recapture analyses

Capture-recapture estimates abundance using the cases identified in each source and their overlap to calculate the number missed by all [18]. We assumed the population was closed and that temporality was present [11, 19]. We combined all administrative data into a single source at the outset, assuming and thereby accounting for dependency [12]. We assessed other dependencies by calculating the probability of being captured in one source given being in another [11]. Additionally, we evaluated random detection using Pearson’s chi-squared tests of the observed versus expected number of cases in a pair of sources [11]. Both were assessed under the prevalence structure to ensure the assumption of independence for these tests was not violated. We used these results to select a dependency structure in combination with theoretical considerations [20]. We evaluated heterogeneity by assessing the linearity of heterogeneity graphs [19].

We used models that accounted for a closed population, temporality, and heterogeneity if determined to be present. The latter was expected as milder, older cases of pertussis are less likely to be tested, have a positive result, and be reported to surveillance [5, 6]. We explicitly built the selected dependency structure into models by including two-way interaction coefficients for sources hypothesized to be dependent [12]. We chose Chao’s lower bound estimator for total sample size for the capture-recapture models, which additionally accounts for dependency [12]. We compared model estimates to those from Darroch models, which further correct for heterogeneity [21]. We rounded estimates of abundance to the nearest whole number. We considered results statistically significant at alpha ≤ 0.05 and we used the Rcapture package in R [19, 22].

### Estimated sensitivity

We calculated sensitivity by dividing the number of cases identified by each data source by the estimated abundance [11]. For the incidence and adjusted false positive case definitions, there was concern that correlation (clustering) would arise from having multiple cases for some individuals, impacting the sensitivity point estimate and variance [23, 24]. To address this, sensitivity was additionally calculated under both definitions by selecting a random episode per person to remove the effect of clustering. We then compared these results to those including multiple episodes, with little difference used as evidence that estimates were robust.

## Results

### Capture-recapture results

**Fig 1:**
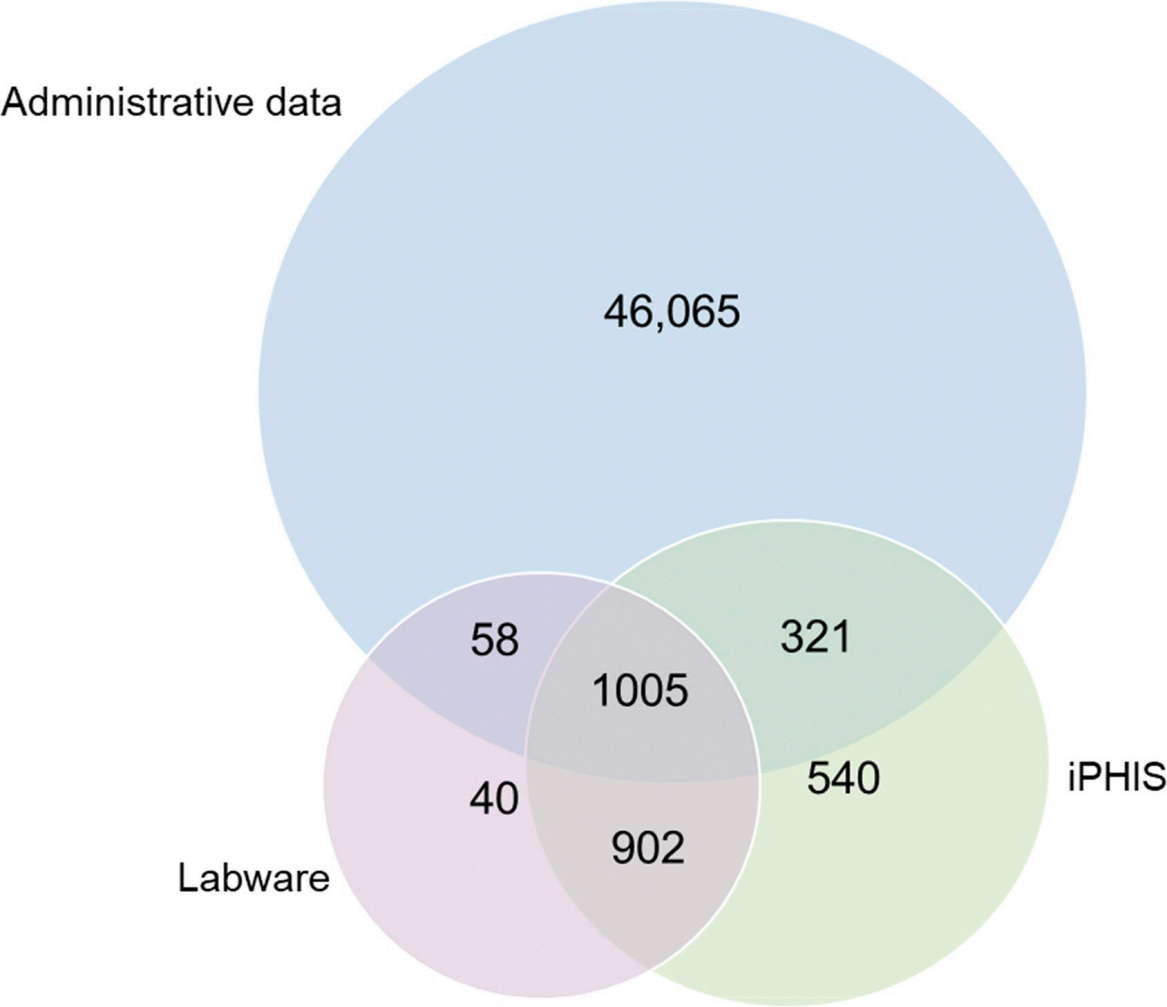
Example of overlap between data sources^Ϯ^, all age groups combined under the period prevalence primary analysis. ^Ϯ^Administrative data = OHIP physician diagnostic billing codes, DAD hospitalizations, NACRS emergency room visits, iPHIS = reportable cases, Labware = laboratory tests.

Under the prevalence case definition, all sources were dependent based on pair-wise probabilities and Pearson’s chi-squared test was highly significant (p < 0.00001), suggesting non-random detection. This provided support for the theorized dependency structure, and we used all two-way interaction terms to account for dependency between each source pair. To ensure comparability between case definitions, we used this structure for all capture-recapture models. A visual depiction of dependency is presented in Fig 1 using the degree of overlap between data sources under prevalence. Heterogeneity graphs lacked linearity, indicating heterogeneity was present and had to be accounted for in modelling (S2 Fig).

Estimated abundance was similar for those less than one year of age under the definitions for period prevalence and incidence with exclusions, at 3810 (95% CI 2932–5707) and 3078 (95% CI 2362–4609) respectively (Table 3). Abundance was considerably lower with less variability, as measured by the width of the 95% CIs, for the adjusted false positive definition at 1151 (95% CI 964–1538). Overall, the one year or older age group had more variable estimates. Abundance for this age group was again similar under prevalence and incidence, at 114,135 (95% CI 87,228–155,391) and 132,528 (95% CI 100,384–181,775). The false positive definition had substantially lower estimates and variability at 20,490 (95% CI 15,998–27,319). Darroch models produced identical abundance estimates in scenarios with more than one two-way interaction term.

**Table 3:** Capture-recapture model results with all two-way interactions between sources by analysis, age group, and case definition.

		Total observed pertussis cases	Chao model with all two-way interactions		
			Estimated abundance	95% CI	AIC, BIC*
**PRIMARY ANALYSIS** ^Ϯ^					
*< 1 year of age*					
	Period prevalence	2636	3810	2932–5707	56.9, 98.1
	Incidence with exclusions	2118	3078	2362–4609	56.8, 96.4
	Adjusted false positives	904	1151	964–1538	55.4, 89.1
*1+ years of age*					
	Period prevalence	46,295	114,135	87,228–155,391	69.9, 131.1
	Incidence with exclusions	48,676	132,528	100,384–181,775	70.1, 131.6
	Adjusted false positives	8828	20,490	15,998–27,319	68.1, 117.7
**SENSITIVITY ANALYSIS 1** ^Ϯ^					
*< 1 year of age*					
	Period prevalence	2648	4030	3003–6240	57.3, 98.5
	Incidence with exclusions	2133	3235	2418–4974	57.1, 96.8
	Adjusted false positives	897	1163	963–1575	55.7, 89.3
*1+ years of age*					
	Period prevalence	46,581	102,954	80,534–136,981	70.8, 132.1
	Incidence with exclusions	49,015	119,684	92,638–160,724	71.1, 132.7
	Adjusted false positives	9295	19,120	15,339–24,804	69.1, 119.0
**SENSITIVITY ANALYSIS 2** ^Ϯ^					
*< 1 year of age*					
	Period prevalence	2729	9197	6180–14,619	60.7, 102.1
	Incidence with exclusions	2206	7883	5205–12,740	60.1, 100.0
	Adjusted false positives	1007	2454	1759–3691	58.8, 93.2
*1+ years of age*					
	Period prevalence	47,257	224,995	187,309–272,926	75.2, 136.5
	Incidence with exclusions	49,551	252,872	209,058–308,891	74.9, 136.6
	Adjusted false positives	9917	37,868	31,802–45,596	72.9, 123.3
**SENSTIVITY ANALYSIS 3** ^Ϯ^					
*< 1 year of age*					
	Period prevalence	2050	3615	2585–5857	57.4, 96.8
	Incidence with exclusions	1863	3249	2341–5192	57.4, 96.1
	Adjusted false positives	626	769	671–963	55.1, 86.2
*1+ years of age*					
	Period prevalence	42,446	171,908	119,993–257,414	69.2, 129.8
	Incidence with exclusions	46,411	207,041	143,834–311,111	69.4, 130.6
	Adjusted false positives	6541	21,678	15,685–31,450	67.0, 114.5

*AIC = Akaike information criterion and BIC = Bayesian information criterion, for both lower values indicate a comparably better model fit

^Ϯ^Primary analysis = includes confirmed iPHIS case reports, Labware positive laboratory tests, and health administrative data, Sensitivity analysis 1 = additionally included probable iPHIS case reports, Sensitivity analysis 2 = additionally included iPHIS probable and “does not meet” case reports and Labware indeterminate laboratory tests, Sensitivity analysis 3 = additionally excluded A37.9 codes (pertussis species unspecified)

### Sensitivity analyses

After the addition of probable iPHIS cases, there was little change to estimated abundance (Table 3). Including iPHIS probable and DNM cases and indeterminate laboratory tests produced the largest increase in abundance and introduced a considerable amount of variability. While this pattern occurred under all case definitions, the highest estimate of 252,872 (95% CI 209,058–308,891) was obtained under incidence for those one year of age or older. After removing A37.9 codes, abundance estimates increased and decreased without a reliable pattern.

### Estimated sensitivity

Sensitivity estimates are available in Table 4. Results were comparable and generally within a few percentage points with and without multiple episodes per individual (S1 Table). As a result, clustering was determined to have a minimal effect and we reported sensitivity estimates that included multiple episodes per individual. Using all data sources consistently provided the highest sensitivity (Table 4). Labware had the lowest sensitivity while administrative data had the highest for a single source. Under the primary analysis and prevalence definition, the highest sensitivity for those less than one year of age was 69.2% (95% CI 67.7–70.7%), which dropped to 40.6% (95% CI 40.3–40.8%) for the older age group. Incidence sensitivity estimates were similar but slightly lower in comparison, excepting marginally higher estimates for iPHIS and Labware for the younger age group. Overall, using the adjusted false positive definition increased sensitivity, with all sources under the primary analysis producing a sensitivity of 78.5% (95% CI 76.2–80.9%) and 43.1% (95% CI 42.4–43.8%) for the younger and older age groups respectively. However, the largest increase to sensitivity occurred for iPHIS and Labware estimates, although sensitivity for these sources remained low for the older age group.

**Table 4:** Estimated sensitivity by case definition, age group, analysis, and data source.

		PERIOD PREVALENCE				INCIDENCE WITH EXCLUSIONS				ADJUSTED FALSE POSITIVES			
Analysis	Data source	Sensitivity (%)		(n/N)^§^	95% CI (%)	Sensitivity (%)		(n/N)^§^	95% CI (%)	Sensitivity (%)		(n/N)^§^	95% CI (%)
**< 1 YEAR OF AGE**													
Primary analysis^Ϯ^	All data sources	69.2	(2636/3810)		67.7–70.7	68.8	(2118/3078)		67.2–70.4	78.5	(904/1151)		76.2–80.9
	Admin data	66.0	(2516/3810)		64.5–67.5	64.8	(1994/3078)		63.1–66.5	67.8	(780/1151)		65.1–70.5
	Labware	9.69	(369/3810)		8.75–10.6	12.0–12.1	(368-372/3078)*		10.8–13.2	32.0–32.3	(368-372/1151)*		29.3–35.0
	iPHIS	12.1	(461/3810)		11.1–13.1	14.8–14.9	(456-460/3078)*		13.7–16.2	39.6–40.0	(456-460/1151)*		36.8–42.8
Sensitivity analysis 1^Ϯ^	All data sources	65.7	(2648/4030)		64.2–67.2	65.9	(2133/3235)		64.3–67.6	77.1	(897/1163)		74.7–79.5
	Admin data	62.4	(2516/4030)		60.9–63.9	61.6–61.7	(1993-1997/3235)		60.1–63.4	65.4	(761/1163)		63.8–67.0
	Labware	9.16	(369/4030)		8.27–10.0	11.4–11.5	(368-372/3235)*		10.3–12.6	31.6–32.0	(368-372/1163)*		30.1–33.2
	iPHIS	11.9	(481/4030)		10.9–12.9	14.7–14.8	(476-480/3235)*		13.5–16.1	40.9–41.3	(476-480/1163)*		39.6–42.9
Sensitivity analysis 2^Ϯ^	All data sources	29.7	(2729/9197)		28.7–30.6	28.0	(2206/7883)		27.0–29.0	41.0	(1007/2454)		39.1–43.0
	Admin data	27.3–27.4	(2515-2519/9197)*		26.4–28.3	25.3–25.4	(1995-1999/7883)*		24.3–26.3	32.6	(800/2454)		30.7–34.5
	Labware	5.09	(468/9197)		4.64–5.54	5.95–6.00	(469-473/7883)*		5.43–6.52	19.1	(469/2454)		17.6–20.7
	iPHIS	5.96	(548/9197)		5.47–6.44	6.56	(517/7883)		6.01–7.10	21.1	(517/2454)		19.5–22.7
Sensitivity analysis 3^Ϯ^	All data sources	56.7	(2050/3615)		55.1–58.3	57.3	(1863/3249)		55.6–59.0	81.4	(626/769)		78.7–84.2
	Admin data	50.7	(1832/3615)		49.0–52.3	50.5	(1642/3249)		48.8–52.3	52.7	(405/769)		49.1–56.2
	Labware	10.2–10.3	(368-372/3615)*		9.19–11.3	11.3–11.4	(368-372/3249)*		10.2–12.5	47.9–48.4	(368-372/769)*		44.3–51.9
	iPHIS	12.8	(461/3615)		11.7–13.8	14.2	(461/3249)		13.0–15.4	59.9	(461/769)		56.5–63.4
**1 + YEARS OF AGE**													
Primary analysis^Ϯ^	All data sources	40.6	(46,295/114,135)		40.3–40.8	36.7	(48,676/132,528)		36.5–37.0	43.1	(8823/20,490)		42.4–43.8
	Admin data	39.4	(44,933/114,135)		39.1–39.7	35.7	(47,292/132,528)		35.4–35.9	36.3	(7444/20,490)		35.7–37.0
	Labware	1.43	(1636/114,135)		1.36–1.50	1.24	(1637-1641/132,528)*		1.18–1.30	7.99–8.01	(1637-1641/20,490)*		7.62–8.38
	iPHIS	2.02	(2307/114,135)		1.94–2.10	1.74	(2305-2309/132,528)*		1.67–1.81	11.2–11.3	(2305-2309/20,490)*		10.8–11.7
Sensitivity analysis 1^Ϯ^	All data sources	45.2	(46,581/102,954)		44.9–45.5	41.0	(49,015/119,684)		40.7–41.2	48.6	(9295/19,120)		47.9–49.3
	Admin data	43.6	(44,931–44,935/102,954)*		43.3–43.9	39.6	(47,339/119,684)		37.9–41.2	39.8	(7619/19,120)		38.2–41.5
	Labware	1.59	(1636/102,954)		1.51–1.67	1.37	(1637-1641/119,684)*		0.98–1.76	8.56–8.58	(1637-1641/19,120)*		7.62–9.53
	iPHIS	2.72	(2800/102,954)		2.62–2.82	2.34	(2798-2802/119,684)*		1.83–2.85	14.6–14.7	(2798-2802/19,120)*		13.5–15.8
Sensitivity analysis 2^Ϯ^	All data sources	21.0	(47,257/224,995)		20.8–21.2	19.6	(49,551/252,872)		19.4–19.8	26.2	(9917/37,868)		25.7–26.6
	Admin data	20.0	(44,926/224,995)		19.8–20.1	18.7	(47,346/252,872)		18.6–18.9	20.4	(7712/37,868)		20.0–20.8
	Labware	1.03	(2318/224,995)		0.99–1.07	0.92	(2318-2322/252,872)*		0.88–0.96	6.12–6.13	(2318-2322/37,868)*		5.88–6.37
	iPHIS	1.48	(3332/224,995)		1.43–1.53	1.22	(3079/252,872)		1.17–1.26	8.13	(3079/37,868)		7.86–8.41
Sensitivity analysis 3^Ϯ^	All data sources	24.7	(42,446/171,908)		24.5–24.9	22.4	(46,411/207,041)		22.2–22.6	30.2	(6541/21,678)		29.6–30.8
	Admin data	23.7	(40,710/171,908)		23.5–23.9	21.6	(44,657/207,041)		21.4–21.7	22.1	(4787/21,678)		20.7–23.5
	Labware	0.95	(1633-1637/171,908)*		0.90–1.00	0.79	(1633-1637/207,041)*		0.75–0.83	7.53–7.55	(1633-1637/21,678)*		6.64–8.44
	iPHIS	1.34–1.35	(2310-2314/171,908)*		1.29–1.40	1.12	(2311-2315/207,041)*		1.07–1.16	10.7	(2311-2315/21,678)*		9.62–11.7

*suppressed for reporting due to low cell size (direct or by inference)

^§^the observed in individual data sources will not sum to the observed in all data sources due to overlap between sources

^Ϯ^Primary analysis = includes confirmed iPHIS case reports, Labware positive laboratory tests, and health administrative data, Sensitivity analysis 1 = additionally included probable iPHIS case reports, Sensitivity analysis 2 = additionally included iPHIS probable and “does not meet” case reports and Labware indeterminate laboratory tests, Sensitivity analysis 3 = additionally excluded A37.9 codes (pertussis species unspecified)

Any change to sensitivity estimates after the addition of probable iPHIS cases was small, within 5%. After including iPHIS probable and DNM cases and indeterminate laboratory tests, the sensitivity estimates for all sources combined were more similar between age groups. Under prevalence, sensitivity was 29.7% (95% CI 28.7–30.6%) and 21.0% (95% CI 20.8–21.2%) for the younger and older groups respectively. Excluding A37.9 codes under the adjusted false positive definition led to the highest Labware and iPHIS sensitivity estimates for the younger age group, at close to 50% and 60%.

## Discussion

Abundance estimates demonstrated that a high proportion of pertussis cases were missed by all sources. Results were similar when using prevalence and incidence, but after adjusting for physician billing code-only false positives abundance dropped considerably. While this occurred for both age groups, the effect was greater among those one year of age or older. The low estimated PPV of physician billing code-only episodes provides evidence that false positives have been inflating observed counts and abundance estimates from capture-recapture analyses of pertussis in Ontario, which decreased sensitivity for laboratory and case report data. As a result, the adjusted case definition is the most valid out of those tested, with the described approach useful for improving the utility of capture-recapture methods to epidemiological surveillance. This procedure details how to incorporate sources of health administrative data to improve passive pertussis surveillance estimates, while accounting for validity issues inherent in such secondary data. In the future, the estimated PPV may be used for adjustment prior to conducting capture-recapture analyses. Alternatively, the validation strategy may be used to calculate unique validity estimates prior to adjustment, with extensions to other diseases and jurisdictions.

Regardless of the case definition, health administrative data had the highest sensitivity for a single source, with all sources combined producing the best sensitivity. This establishes the value of incorporating health administrative data into pertussis surveillance if accuracy and timeliness limitations are addressed. Laboratory tests had the lowest sensitivity and particularly for the older age group, indicating testing bias is present. Public health case reports had slightly higher sensitivity but displayed a similar pattern. While sensitivity estimates improved for both after adjusting for false positives, this was primarily in the younger age group. Overall, sensitivity was substantially lower for the older group, suggesting ascertainment bias is present.

A 2018 Ontario capture-recapture study used the same data sources but over fewer years. As a result, abundance is not directly comparable as the higher estimates from this study are expected due to the extra years of data. However, sensitivity for all sources with probable case reports included was estimated at 54% and 39% for the younger and older age groups respectively. This is comparable to sensitivity estimates for the older age group in this study after including iPHIS probable cases, but sensitivity was higher for the younger group (~66%). This could be due to improved recapture in this subgroup using the case definitions or differences in how the 2018 study modelled dependencies [11]. The 2018 study reported considerable uncertainty persisting around physician billing code accuracy and investigated by using different proportions of true positives for all health administrative data, with the lowest at 25%. This dropped abundance estimates by 66% and 73% for the younger and older age groups [11]. We were able to address remaining uncertainty through validation of physician billing code-only episodes. Interestingly, applying the estimated PPV of 8.99% to these episodes reduced abundance estimates similarly to assuming a PPV of 25% for all health administrative data in the 2018 study, by 64% and 84%. The higher latter value indicates a greater proportion of code only-episodes in the older age group, leading to enhanced improvement in recapture once removed. Laboratory test and case report sensitivity considerably improved using this case definition.

A modelling study based on pertussis incidence in southern Ontario from 1993–2004 estimated that five to 33,032 cases remain undetected per reported case depending on age [4]. It has been stated elsewhere that the true number of pertussis cases is at least three times higher than what is reported [3]. To compare, estimates from this study should be calculated using the false positive case definition to avoid inflating underdetection. For case report data, these values are 2.5 and 8.9 for the younger and older age groups under the primary analysis and 4.7 and 12 after including probable and DNM case reports and indeterminate laboratory tests. Using all data sources, 1.3 and 2.3 cases were missed per observed case for the younger and older age groups, which increased to 2.4 and 3.8 with the additional case reports and laboratory tests. The substantially lower upper limit compared to the modelling study is likely due to differences in age groups, pertussis incidence during the respective time periods, and diagnostic testing accuracy, with PCR introduced after 2004. In addition, the estimation approaches differ, with the modelling study using methods with considerable uncertainty as reflected by the wide range of values. While this study used the more conservative Chao’s lower bound estimator, similar results were obtained from Darroch models. Furthermore, a simulation study found that Chao’s methods estimated abundance within 75–82% of the total population size in most complicated scenarios [12]. Regardless, this study’s findings are in line with past estimates of underdetection, with reasonable explanations for remaining differences.

The estimated PPV for physician billing code-only episodes of 8.99% (95% CI 1.59–16.39%) is comparable to the PPV of 13.6% (95% CI 9.28–17.9%) obtained for an OHIP physician billing code algorithm within the EMRPC using the same cohort [17]. The slightly higher PPV in the EMRPC can be explained by using prevalent cases, increasing the likelihood of concordance between codes and cases. Additionally, EMRPC billing codes were only collected from physician offices, potentially decreasing the number of false positives. OHIP pertussis cases billed at homes or long-term care facilities are unlikely to be documented in EMRPC’s primary care patient records. Further contributing to this issue is that visits outside office settings such as walk-in or specialist visits fail to be captured in the EMRPC, leading to about 15% of interactions being missed [25]. In addition, only two thirds of the laboratory tests in OHIP are documented in the EMRPC [25]. Missing any of these data in the EMRPC could artificially decrease the PPV of OHIP billing code-only episodes by increasing the number of false positives, although billings from outside office settings were uncommon in the OHIP database and unlikely to greatly affect validation. The EMRPC study only tested the accuracy of data available in the EMRPC, meaning sensitivity estimates for emergency room visits, hospitalizations, and case report data are not available. However, pertussis laboratory test sensitivity using prevalent cases was reported as 0.64% (95% CI 0.37–1.09%) across all ages [17]. After adjusting for false positives, 8.0% sensitivity (95% CI 7.62–8.38%) was obtained for the older age group (which only excludes infants) using prevalence. This difference can be explained by variation in ages, pertussis classification, and validation methods. Additionally, Labware has more comprehensive coverage, with the EMRPC noted to have decreased laboratory test sensitivity through incomplete documentation [17].

One limitation of this study is that we did not validate physician billing code-only episodes separately for the younger and older age groups. This was to preserve an adequate sample size, with the assumption that the PPV averages out across age groups and this is an appropriate strategy for taking a random sample based on a proportion. In addition, while demonstrating that sensitivity estimates differ by age group, it is unlikely that PPV would vary to the same extent. PPV evaluates the proportion of true positives out of test positives and is primarily affected by prevalence, not testing bias [26]. Although it may appear that younger individuals have a higher risk of pertussis infection, due to testing and ascertainment bias this may not actually be the case [5]. Older cases are hypothesized to be an important source of pertussis, which is evident through older relatives being key sources of transmission to infants [5, 27]. Additionally, in 2019, 62% of reported cases in Ontario occurred in those ten years of age or older [28]. However, it may be of interest to allow the PPV to vary separately by age group in future analyses.

An additional limitation is that we had to remove EMRPC cases without dates during validation. We considered it preferable to avoid introducing misclassification rather than preserving the sample size, and there is no reason to suspect excluded cases are systematically different from the majority of those included. A further limitation of validation is that we were unable to adjust the PPV and sensitivity estimates for clustering under the incidence and false positive definitions due to the low sample size and study methodology respectively [23, 24]. To address this, we compared point estimates and variances to those using a single episode per individual to assess the effect of correlation, with little difference found between PPV estimates. While some sensitivity estimates were statistically significantly different, the absolute difference was small, and this was for the older age group where large sample sizes produced substantial precision. As a result, we concluded that these differences were unlikely to be clinically significant. While it is possible correlation still minorly affected the findings, it is a study strength to be able to report sensitivity estimates under different case definitions. Little validation research has incorporated repeated episodes, which is of interest for acute diseases.

Finally, missing data may impact abundance and sensitivity estimates, but this is an inherent limitation of using secondary data and beyond the scope of this study. This includes failing to collect data on certain cases, such as those that are milder in nature who do not seek health care or may be misdiagnosed. While pertussis infectivity is related to severity, meaning that milder undetected cases are likely less infectious, it is possible that many are still important to transmission. While Labware data only covers pertussis laboratory testing for < 95% of Ontario, we assumed that the remaining tests would not considerably impact abundance estimates [11, 29]. To assess the effect of missing data, we conducted sensitivity analyses incorporating additional data to determine the robustness of capture-recapture abundance estimates. This produced little change to results, except when including probable and DNM cases in addition to indeterminate laboratory tests. Doing so decreased sensitivity and led to greater similarity in capture patterns between the younger and older age groups. This could suggest that older individuals are less likely to meet the confirmed case definition, or that milder infections are frequently missed in younger as well as older individuals. Alternatively, it could be due to increased uncertainty in abundance among both groups under this analysis, stemming from greater uncertainty in true pertussis status.

## Conclusions

This study demonstrated how limited health data accuracy can be accounted for using capture-recapture analyses that employ different pertussis case definitions. The false-positive adjusted case definition helped address past uncertainty in burden estimation and produced results which align with the degree of underdetection reported in the literature. The described methodology allows passive pertussis case report data to be supplemented with health administrative data to better estimate burden, while accounting for validity issues that accompany secondary data use. Improved capture-recapture estimates can better inform public health policy and prevention. Findings consistently demonstrated that data sources are failing to detect pertussis cases, and particularly laboratory and case report data. The best sensitivity was obtained by using all sources together, with health administrative data having the highest sensitivity for a single source. This indicates the benefit of incorporating real time health administrative data into surveillance if misclassification can be addressed. The results provide further support that pertussis detection differs by age, indicating that ascertainment and testing bias is present in data.

## Supporting information

S1 Fig Rules for developing incident case episodes and establishing data re-capture. (TIF)

S2 Fig Heterogeneity graphs from the primary analysis by case definition and age group. (TIF)

S1 Table Estimated sensitivity by age group, analysis, and data source for incidence and adjusted false positive case definitions using a single random episode per person. (DOCX)

S1 Appendix Validation of Ontario Health Insurance Plan (OHIP) billing diagnostic code-only case episodes. (DOCX)

## References

[1] Public Health Agency of Canada. Reported cases from 1924 to 2019 in Canada–Notifiable diseases on-line; [updated 2021 July 20; cited 2022 March 3]. Database: Government of Canada [Internet]. Available from: http://diseases.canada.ca/notifiable/charts?c=pl.

[2] Savage R, Whelan M, Johnson I, Rhea E, LaFreniere M, Rosella LC, et al. Assessing secondary attack rates among household contacts at the beginning of the influenza A (H1N1) pandemic in Ontario, Canada, April-June 2009: A prospective, observational study. BMC Public Health. 2011;11(1):234. doi: 10.1186/1471-2458-11-234 21492445 PMC3095560

[3] Kilgore PE, Salim AM, Zervos MJ, Schmitt H-J. Pertussis: Microbiology, disease, treatment, and prevention. Clin Microbiol Rev. 2016;29(3): 449–486. doi: 10.1128/CMR.00083-15 27029594 PMC4861987

[4] McGirr AA, Tuite AR, Fisman DN. Estimation of the underlying burden of pertussis in adolescents and adults in Southern Ontario, Canada. PLoS One. 2013;8(12): e83850. doi: 10.1371/journal.pone.0083850 24376767 PMC3871538

[5] Crowcroft N, Miller E. Pertussis epidemiology. In: Rohani P, Scarpino S, editors. Pertussis: Epidemiology, immunology, and evolution. Oxford, UK: Oxford University Press; 2019. pp. 66–86.

[6] Deeks S, De Serres G, Boulianne N, Duval B, Rochette L, Dery P, et al. Failure of physicians to consider the diagnosis of pertussis in children. Clin Infect Dis. 1999;28(4): 840–846. doi: 10.1086/515203 10825048

[7] Bolotin S, Quinn H, McIntyre P. Surveillance and diagnostics. In: Rohani P, Scarpino S, editors. Pertussis: Epidemiology, immunology, and evolution. Oxford, UK: Oxford University Press; 2019. pp. 193–210.

[8] World Health Organization. Pertussis: Vaccine-Preventable Diseases Surveillance Standards. [Internet]. WHO; 2018. Available from: https://www.who.int/publications/m/item/vaccine-preventable-diseases-surveillance-standards-pertussis.

[9] Barkoff AM, Grondahl-Yli-Hannuksela K, He Q. Seroprevalence studies of pertussis: What have we learned from different immunized populations. Pathog Dis. 2015;73(7): ftv050. doi: 10.1093/femspd/ftv050 26208655

[10] Crowcroft NS, Stein C, Duclos P, Birmingham M. How best to estimate the global burden of pertussis? Lancet Infect Dis. 2003;3(7): 413–418. doi: 10.1016/s1473-3099(03)00669-8 12837346

[11] Crowcroft NS, Johnson C, Chen C, Li Y, Marchand-Austin A, Bolotin S, et al. Under-reporting of pertussis in Ontario: A Canadian Immunization Research Network (CIRN) study using capture-recapture. PLoS One. 2018;13(5): e0195984. doi: 10.1371/journal.pone.0195984 29718945 PMC5931792

[12] Braeye T, Verheagen J, Mignon A, Flipse W, Pierard D, Huygen K, et al. Capture-recapture estimators in epidemiology with applications to pertussis and pneumococcal invasive disease surveillance. PLoS One. 2016;11(8): e0159832. doi: 10.1371/journal.pone.0159832 27529167 PMC4987016

[13] Böhning D, Rocchetti I, Maruotti A, Holling H. Estimating the undetected infections in the Covid-19 outbreak by harnessing capture-recapture methods. Int J Infect Dis. 2020;97: 197–201. doi: 10.1016/j.ijid.2020.06.009 32534143 PMC7286831

[14] Polonsky JA, Böhning D, Keita M, Ahuka-Mundeke S, Nsio-Mbeta J, Abedi AA, et al. Novel use of capture-recapture methods to estimate completeness of contact tracing during an Ebola outbreak, Democratic Republic of the Congo, 2018–2020. Emerg Infect Dis. 2021;27(12): 3063–3072. doi: 10.3201/eid2712.204958 34808076 PMC8632194

[15] Crowcroft NS, Andrews N, Rooney C, Brisson M, Miller E. Deaths from pertussis are underestimated in England. Arch Dis Child. 2002;86(5): 336–338. doi: 10.1136/adc.86.5.336 11970924 PMC1751114

[16] McBurney SH, Kwong JC, Brown KA, Rudzicz F, Chen B, Candido E, et al. Developing a reference standard for pertussis by applying a stratified sampling strategy to electronic medical record data. Ann Epidemiol. 2023;77:53–60. doi: 10.1016/j.annepidem.2022.11.002 36372292

[17] McBurney SH, Kwong JC, Brown KA, Rudzicz F, Chen B, Candido E, et al. Validating pertussis data measures using electronic medical record data in Ontario, Canada 1986–2016. In: McBurney SH. The problem with pertussis: Finding undetected pertussis cases in Electronic Medical Record Primary Care to improve data accuracy and burden estimates. PhD. Thesis, The University of Toronto. 2022. Available from: https://tspace.library.utoronto.ca/handle/1807/9945.

[18] Buehler JW. Surveillance. In: Rothman KJ, Greenland S, Lash TL, editors. Modern epidemiology. 3rd ed. Philadelphia: Wolters Kluwer Health/Lippincott Williams & Wilkins; 2008. pp. 459–480.

[19] Baillargeon S, Rivest L-P. Rcapture: Loglinear models for capture-recapture in R. J Stat Softw. 2007;19(5): 1–31. doi: 10.18637/jss.v019.i0521494410

[20] Jones HE, Hickman M, Welton NJ, De Angelis D, Harris RJ, Ades AE. Recapture or precapture? Fallibility of standard capture-recapture methods in the presence of referrals between sources. Am J Epidemiol. 2014;179(11): 1383–1393. doi: 10.1093/aje/kwu056 24727806 PMC4036210

[21] Rivest L-P, Baillargeon S. Applications and extensions of Chao’s moment estimator for the size of a closed population. Biometrics. 2007;63(4): 999–1006. doi: 10.1111/j.1541-0420.2007.00779.x 17425635

[22] R-Core-Team. R: A language and environment for statistical computing. Vienna (AT): R Foundation for Statistical Computing; 2013.

[23] Ying GS, Maguire MG, Glynn RJ, Rosner B. Calculating sensitivity, specificity, and predictive values for correlated eye data. Investig Ophthalmol Vis Sci. 2020;61(11): 29. doi: 10.1167/iovs.61.11.29 32936302 PMC7500131

[24] Genders TS, Spronk S, Stijnen T, Steyerberg EW, Lesaffre E, Hunink MG. Methods for calculating sensitivity and specificity of clustered data: A tutorial. Radiology. 2012;265(3): 910–916. doi: 10.1148/radiol.12120509 23093680

[25] Tu K, Mitiku T, Ivers NM, Guo H, Lu H, Jaakkimainen L. Evaluation of Electronic Medical Record Administrative data Linked Database (EMRALD). Am J Manag Care. 2014;20: e15–21. 24669409

[26] Molinaro AM. Diagnostic tests: How to estimate the positive predictive value. Neurooncol Pract. 2015;2(4): 162–166. doi: 10.1093/nop/npv030 31386059 PMC6664615

[27] Ministry of Health and Long-Term Care. Infectious Diseases Protocol—Appendix A: Disease-Specific Chapters, Pertussis (Whooping Cough). Government of Ontario; 2019.

[28] Ontario Agency for Health Protection and Promotion. Infectious Disease Trends in Ontario; 2021 [cited 2022 March 3]. Database: Public Health Ontario [Internet]. Available from: https://www.publichealthontario.ca/en/data-and-analysis/infectious-disease/reportable-disease-trends-annually#/42.

[29] Schwartz KL, Kwong JC, Deeks SL, Campitelli MA, Jamieson FB, Marchand-Austin A, et al. Effectiveness of pertussis vaccination and duration of immunity. Can Med Assoc J. 2016;188(16): E399–e406. doi: 10.1503/cmaj.160193 27672225 PMC5088088

