# Supplementary figures and images for: Improving estimates of pertussis burden in Ontario, Canada 2010–2017 by combining validation and capture-recapture methodologies
Source: PLoS One. 2023 Dec 1;18(12):e0273205. doi: 10.1371/journal.pone.0273205 (PMC10691704; doi:10.1371/journal.pone.0273205)

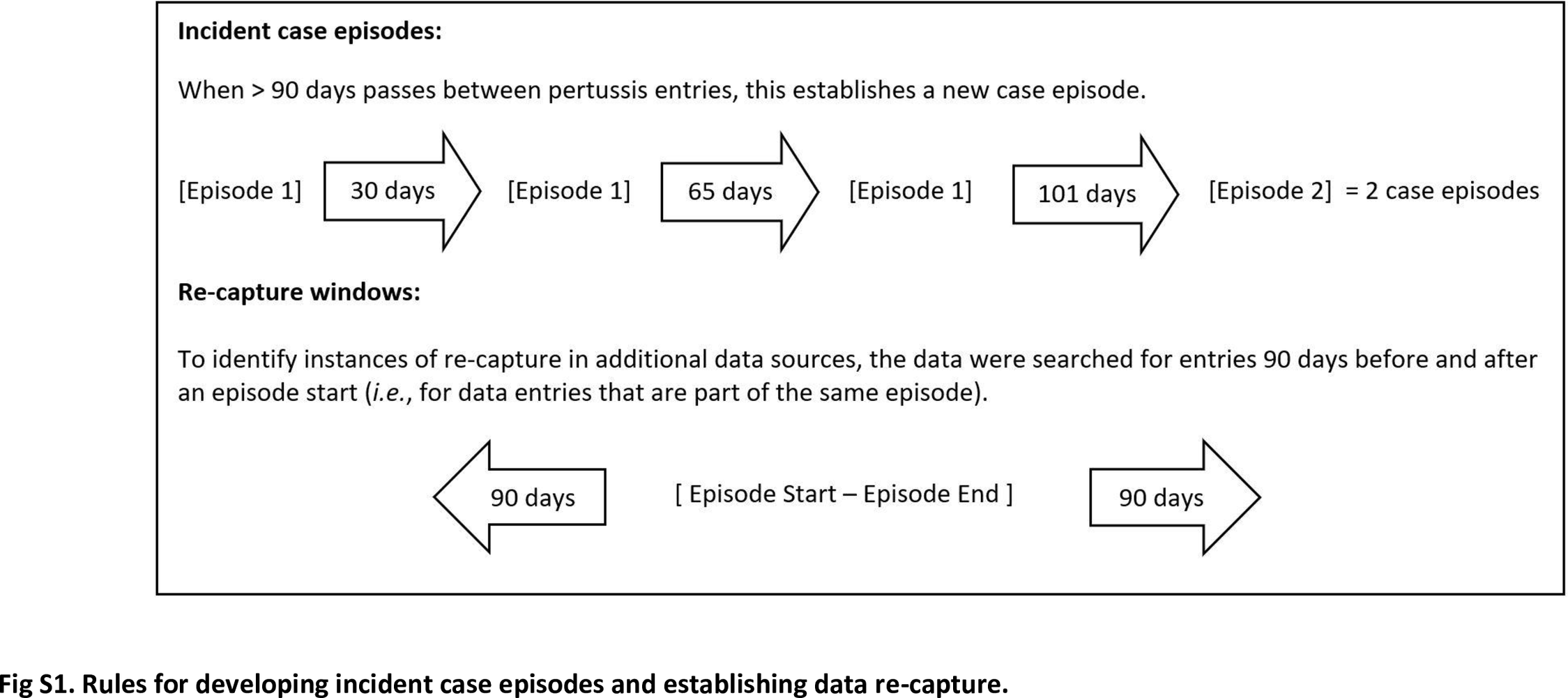

Supplement: S1 Fig — (TIF) [file pone.0273205.s001.tif]

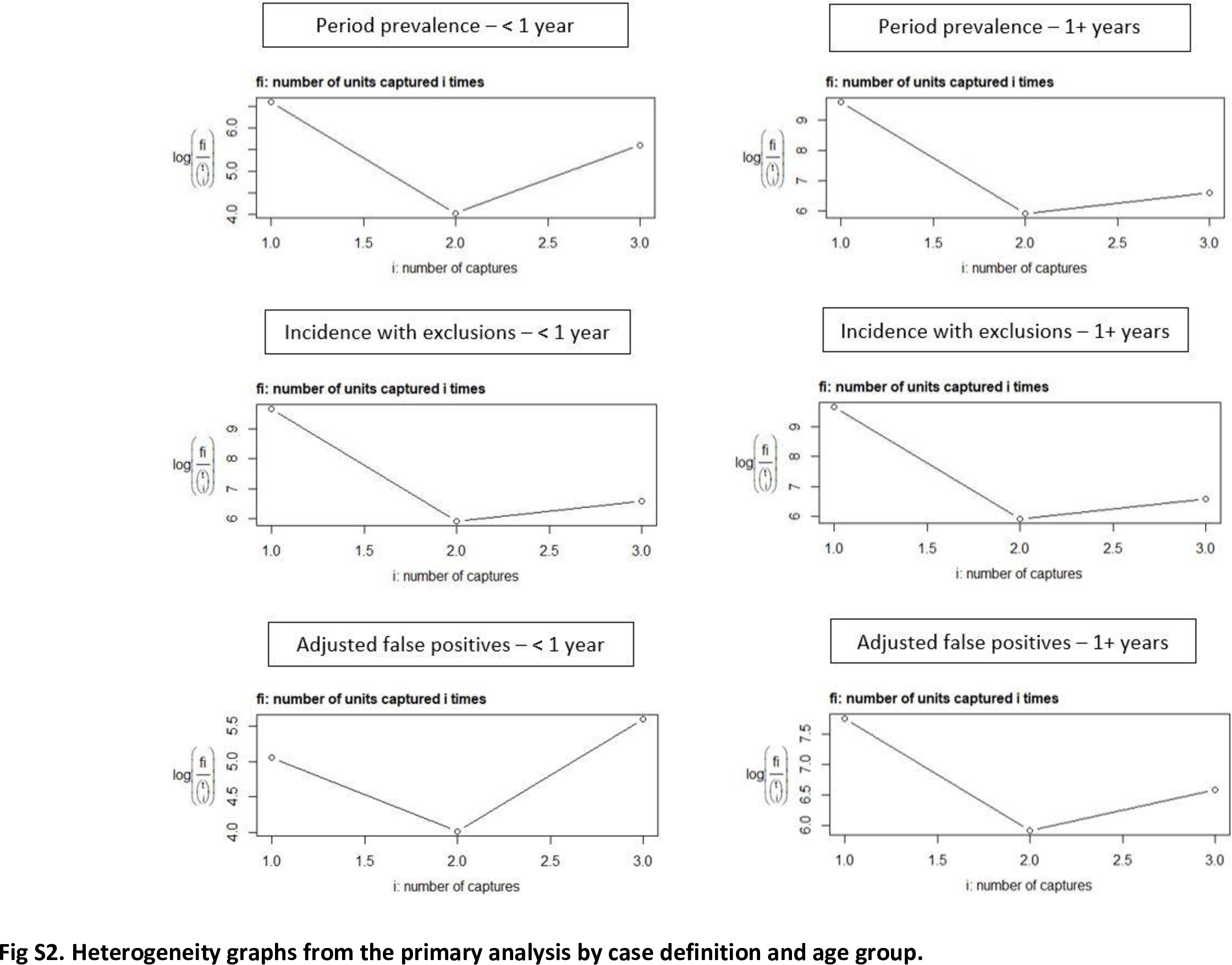

Supplement: S2 Fig — (TIF) [file pone.0273205.s002.tif]
