# Supplementary material for: Improving estimates of pertussis burden in Ontario, Canada 2010–2017 by combining validation and capture-recapture methodologies
Source: PLoS One. 2023 Dec 1;18(12):e0273205. doi: 10.1371/journal.pone.0273205 (PMC10691704; doi:10.1371/journal.pone.0273205)
Supplement: S1 Table — (DOCX) [file pone.0273205.s003.docx]

**Table S1. Estimated sensitivity by age group, analysis, and data source for incidence and adjusted false positive case definitions using a single random episode per person.**

|  | | **INCIDENCE WITH EXCLUSIONS** | | **ADJUSTED FALSE POSITIVES** | |
| --- | --- | --- | --- | --- | --- |
| **Analysis** | **Data source** | **Sensitivity (%)** | **95% CI (%)** | **Sensitivity (%)** | **95% CI (%)** |
| ***< 1 YEAR OF AGE*** | | | | | |
| *Primary analysis* | All data sources | 69.6 | 68.0-71.3 | 77.5 | 75.1-79.9 |
|  | OHIP | 65.4 | 63.7-67.1 | 66.9 | 64.2-69.6 |
|  | Labware | 12.6 | 11.4-13.8 | 31.6 | 28.9-34.3 |
|  | iPHIS | 15.7 | 14.4-17.0 | 39.6 | 36.8-42.4 |
| *Sensitivity analysis 1* ^Ϯ^ | All data sources | 66.8 | 65.1-68.5 | 76.1 | 73.7-78.5 |
|  | OHIP | 62.4 | 60.6-64.1 | 64.5 | 61.8-67.3 |
|  | Labware | 12.0 | 10.9-13.2 | 31.2 | 28.6-33.9 |
|  | iPHIS | 15.7 | 14.4-17.0 | 40.8 | 38.0-43.6 |
| *Sensitivity analysis 2* ^Ϯ^ | All data sources | 28.5 | 27.6-29.5 | 42.0 | 40.0-44.0 |
|  | OHIP | 25.8 | 24.9-26.7 | 33.4 | 31.5-35.3 |
|  | Labware | 6.30 | 5.80-6.79 | 19.6 | 18.0-21.2 |
|  | iPHIS | 6.93 | 6.41-7.45 | 21.6 | 19.9-23.2 |
| *Sensitivity analysis 3* ^Ϯ^ | All data sources | 61.9 | 60.2-63.7 | 84.0 | 81.4-86.6 |
|  | OHIP | 54.3 | 52.4-56.1 | 54.4 | 50.9-58.0 |
|  | Labware | 12.8 | 11.5-14.0 | 49.5 | 45.9-53.1 |
|  | iPHIS | 16.0 | 14.6-17.3 | 61.8 | 58.3-65.3 |
| ***1 + YEARS OF AGE*** | | | | | |
| *Primary analysis* | All data sources | 39.4 | 39.1-39.7 | 45.0 | 44.3-45.7 |
|  | OHIP | 38.2 | 37.9-38.5 | 37.9 | 37.3-38.6 |
|  | Labware | 1.47 | 1.40-1.54 | 8.39 | 8.00-8.78 |
|  | iPHIS | 2.07 | 1.99-2.16 | 11.8 | 11.4-12.3 |
| *Sensitivity analysis 1* ^Ϯ^ | All data sources | 41.2 | 40.9-41.5 | 51.5 | 50.8-52.3 |
|  | OHIP | 39.7 | 39.4-40.0 | 42.2 | 41.5-42.9 |
|  | Labware | 1.52 | 1.44-1.59 | 9.13 | 8.71-9.55 |
|  | iPHIS | 2.60 | 2.50-2.69 | 15.7 | 15.1-16.2 |
| *Sensitivity analysis 2* ^Ϯ^ | All data sources | 20.1 | 19.9-20.2 | 26.9 | 26.4-27.3 |
|  | OHIP | 19.1 | 18.9-19.3 | 20.9 | 20.5-21.3 |
|  | Labware | 1.04 | 0.99-1.08 | 6.31 | 6.06-6.56 |
|  | iPHIS | 1.38 | 1.33-1.43 | 8.40 | 8.11-8.68 |
| *Sensitivity analysis 3* ^Ϯ^ | All data sources | 24.5 | 24.3-24.7 | 31.2 | 30.6-31.9 |
|  | OHIP | 23.5 | 23.3-23.7 | 22.8 | 22.2-23.4 |
|  | Labware | 0.96 | 0.91-1.01 | 7.88 | 7.52-8.25 |
|  | iPHIS | 1.36 | 1.31-1.42 | 11.1 | 10.7-11.6 |

^Ϯ^sensitivity analysis 1 = including probable iPHIS case reports, sensitivity analysis 2 = including iPHIS probable and “does not meet” case reports and Labware indeterminate laboratory tests, sensitivity analysis 3 = removing A37.9 codes with pertussis species unspecified
