# Supplementary material for: Improving estimates of pertussis burden in Ontario, Canada 2010–2017 by combining validation and capture-recapture methodologies
Source: PLoS One. 2023 Dec 1;18(12):e0273205. doi: 10.1371/journal.pone.0273205 (PMC10691704; doi:10.1371/journal.pone.0273205)
Supplement: S1 Appendix — (DOCX) [file pone.0273205.s004.docx]

# **S1 Appendix. Validation of Ontario Health Insurance Plan (OHIP) billing diagnostic code-only case episodes.**

To evaluate the accuracy of OHIP diagnostic code-only episodes, we linked them to a previously developed cohort from the Electronic Medical Record Primary Care (EMRPC) database [1]. EMRPC is one of the only Canadian sources of EMR data available for secondary use to support research and contains patients from over 350 practicing primary care physicians who use PS Suite [2-4]. These patients are representative of the general Ontario population [4]. All 404,922 cohort participants had a data entry in the EMRPC on or after January 1, 1986, with the study period ending on December 30, 2016. This cohort includes a previously developed reference standard of 800 individuals sampled using a stratified strategy and classified as definite pertussis, possible pertussis, ruled-out pertussis, or no mention of pertussis based on record review from two trained abstractors after incorporating laboratory and immunization data [1].

After linkage, we restricted the validation study period to March 7, 2010 until March 30, 2017 to account for data availability to ensure accurate detection between sources (Fig S3). We applied exclusion criteria to the cohort, excluding the reference standard, to ensure EMRPC patients were active on the EMR during the study period and started on the EMR prior to their OHIP episodes (Fig S3). We linked cases and non-cases from the reference standard to the OHIP episodes separately to apply different exclusion criteria. To count as a true positive, an OHIP episode had to occur within 90 days of an EMRPC case episode. We removed EMRPC cases without dates as their timing could not be established (Fig S3). We considered episodes occurring before the study period to be non-cases during the period, with cases with an episode end date less than 90 days before March 7, 2010 removed to create a wash-out period. We applied similar exclusion criteria to non-cases as the rest of the cohort (Supplementary Figure S5.3). We recombined cases, non-cases, and the cohort after data preparation.

## **OHIP code-only episode validation**

A positive predictive value (PPV) was estimated using definite classifications as cases and other classifications as non-cases [5]. We used Begg and Greenes’ method to avoid introducing partial verification bias through the reference standard sampling strategy [6-10]. We used the average PPV or NPV for strata where an OHIP episode was present but not selected for verification, with one person considered verified for calculating within-stratum variance. Further details on this methodology are available elsewhere [5]. To address the possible correlation introduced by having multiple episodes occur for a single individual (clustered data), we calculated a second PPV using a single random episode per person. The resulting PPV (9.55%) was similar to the PPV for all episodes (8.99%), so we concluded that clustering did not substantially impact the point estimate. The variance was also similar, at 0.14 (95% CI 1.59-16.39%) and 0.15 (95% CI 1.89-17.22%) for multiple and single episodes respectively. Combined with the small sample size leading to unstable results, we decided there would be little additional value in reporting a robust standard error to account for the effect of clustering on variance [11]. As a result, clustering was determined to have a minimal effect and we reported estimates that included multiple episodes per individual.


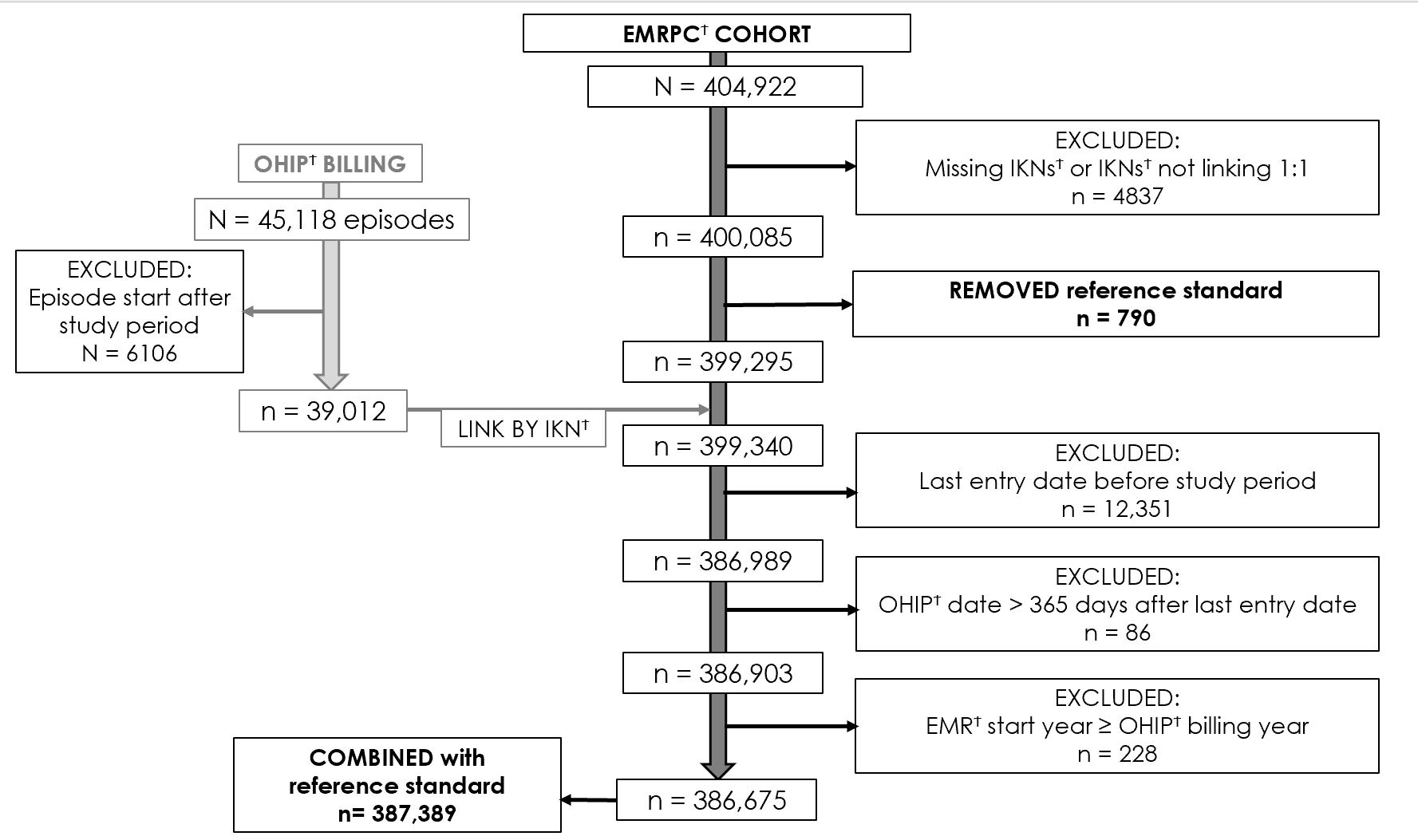


**
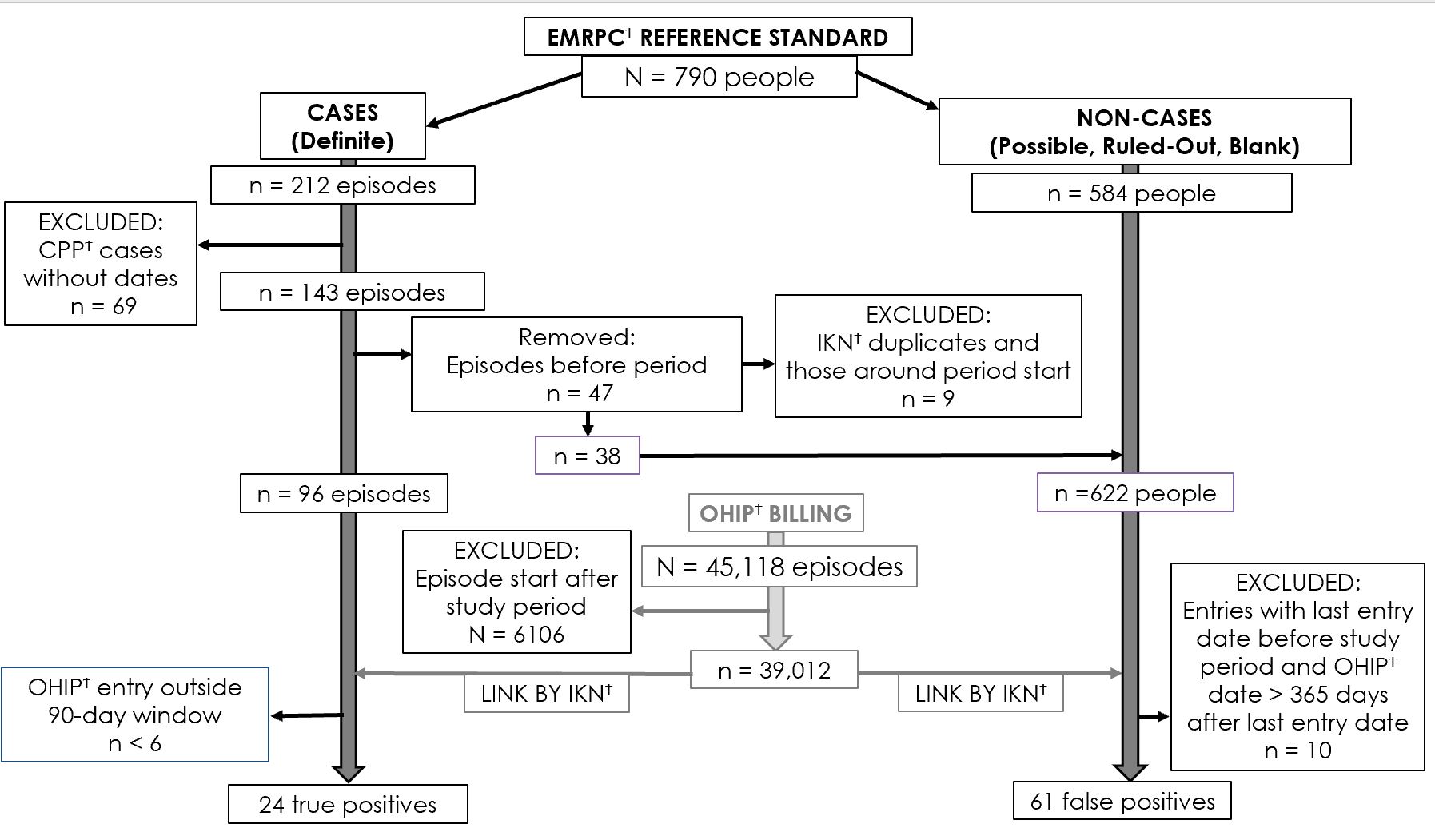
**

**Fig S3. Rules for developing incident case episodes and establishing data re-capture.** ^Ϯ^EMRPC = Electronic Medical Record Primary Care database, OHIP = physician diagnostic billing codes, IKN = unique patient identifier, CPP = cumulative patient profile containing medical history.

## **References**

1. McBurney SH, Kwong JC, Brown KA, Rudzicz F, Chen B, Candido E, et al. Developing a reference standard for pertussis by applying a stratified sampling strategy to electronic medical record data. Ann Epidemiol. 2023;77:53-60. doi: 10.1016/j.annepidem.2022.11.00

2. Wilson SE, Chung H, Schwartz KL, Guttmann A, Deeks SL, Kwong JC, et al. Rotavirus vaccine coverage and factors associated with uptake using linked data: Ontario, Canada. PLoS One. 2018;13(2):e0192809. doi: 10.1371/journal.pone.0192809.

3. Tu K, Mitiku T, Ivers NM, Guo H, Lu H, Jaakkimainen L. Evaluation of Electronic Medical Record Administrative data Linked Database (EMRALD). Am J Manag Care. 2014;20:e15-21.

4. Tu K, Widdifield J, Young J, Oud W, Ivers NM, Butt DA, et al. Are family physicians comprehensively using electronic medical records such that the data can be used for secondary purposes? A Canadian perspective. BMC Medical Inform Decis Mak. 2015;15. doi: 10.1186/s12911-015-0195-x.

5. McBurney SH, Kwong JC, Brown KA, Rudzicz F, Chen B, Candido E, et al. Validating pertussis data measures using electronic medical record data in Ontario, Canada 1986-2016. In: McBurney SH. The problem with pertussis: Finding undetected pertussis cases in Electronic Medical Record Primary Care to improve data accuracy and burden estimates. PhD. Thesis, The University of Toronto. 2022. Available from: https://tspace.library.utoronto.ca/handle/1807/9945

6. Umemneku Chikere CM, Wilson K, Graziadio S, Vale L, Allen AJ. Diagnostic test evaluation methodology: A systematic review of methods employed to evaluate diagnostic tests in the absence of gold standard - An update. PLoS One. 2019;14(10):e0223832. doi: 10.1371/journal.pone.0223832.

7. Cadieux G, Tamblyn R, Buckeridge DL, Dendukuri N. Validation of diagnostic groups based on health care utilization data should adjust for sampling strategy. Med Care. 2017;55(8):e59-e67. doi: 10.1097/MLR.0000000000000324.

8. Begg CB, Greenes RA. Assessment of diagnostic tests when disease verification is subject to selection bias. Biometrics. 1983;39(1):207-215. doi: 10.2307/2530820.

9. Pepe MS. The Statistical Evaluation of Medical Tests for Classification and Prediction. New York, NY: Oxford University Press. 2003.

10. Cronin AM, Vickers AJ. Statistical methods to correct for verification bias in diagnostic studies are inadequate when there are few false negatives: A simulation study. BMC Med Res Methodol. 2008;8(75). doi: 10.1186/1471-2288-8-75.

11. Genders TS, Spronk S, Stijnen T, Steyerberg EW, Lesaffre E, Hunink MG. Methods for calculating sensitivity and specificity of clustered data: A tutorial. Radiology. 2012;265(3):910-916. doi: 10.1148/radiol.12120509.
